# Synthetic Dye - Inorganic Salt Hybrid Colorants for Application in Thermoplastics

**DOI:** 10.3390/molecules16065035

**Published:** 2011-06-17

**Authors:** Yan-Ping Wei, Tian Li, Hong-Wen Gao

**Affiliations:** State Key Laboratory of Environmental Pollution and Resourse Reuse, Tongji University, Shanghai 200092, China; E-Mails: 2009anna_wei@tongji.edu.cn (Y.-P.W.); tianli@tongji.edu.cn (T.L.)

**Keywords:** synthetic dye, hybrid, insoluble inorganic salt, colorant, plastic

## Abstract

Common synthetic dyes, e.g., Weak Acid Pink Red B (APRB, C.I. 18073), Mordant Blue 9 (MB, C.I.14855) and Acid Brilliant Blue 6B (ABB6B, C.I. 42660), can be removed from water by *in situ* hybridization with CaCO_3_, BaSO_4_ and Ca_3_(PO_4_)_2_ and the resulting hybrids thus prepared used as plastic colorants. All the hybrids can be processed into polypropylene (PP) at 200 °C with good color intensity, color brightness and homogeneous dispersion. The BaSO_4_-MB hybrid exhibits better migration resistance to acid and alkali, and stronger covering power than the BaSO_4_-MB mixture. The thermal stability and UV resistance of the Ca_3_(PO_4_)_2_-ABB6B hybrid are better than those of the Ca_3_(PO_4_)_2_-ABB6B mixture. The crystallinity of PP is enhanced by incorporation of these hybrids and the use of these hybrids as colorants in PP instead of the dyes alone is determined to be feasible.

## 1. Introduction

**Figure 1:**
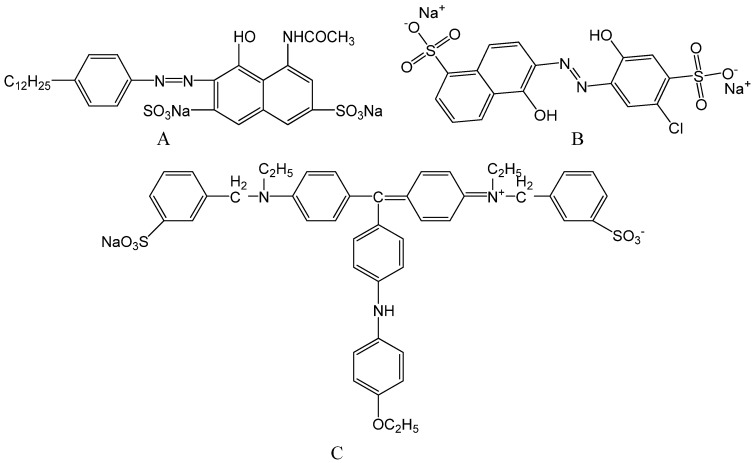
Molecular structures for APRB (**A**), MB (**B**) and ABB6B (**C**).

Colorants include pigments and dyes. Inorganic pigments such as cadmium yellow, iron red, titanium white and copper green are often used as colorants to color plastic and rubber [1], but they lack good light transmission and they also release toxic heavy metals in acidic media, which limits the availability of colours in some applications [2,3]. Organic pigments have been extensively used in paint, ink, and plastic products for their advantages in photosensitivity [4], color strength and excellent transparency, but they also have low coverage, poor dispersion, and especially poor weather durability [5]. Similarly, the over 10,000 commercial organic dyes cover a broad color spectrum [6], but exhibit poor endurance to heat, UV irradiation and solvents, preventing their application in coloring plastics and rubber. Some methods to improve the properties of colorants based on organic dyes have been tried. For example, organic dyes combined with inorganic materials show improved endurance [5,7]. Inspired by the hybridization of waste dyes with inorganic salts [8,9,10], in this work organic/inorganic hybridization was tried to prepare colorants. Calcium carbonate, barium sulfate and calcium phosphate were selected as inorganic supports in this research. There are numerous azo pigments containing Ca and Ba salts of sulphonic acids with structures that are fairly similar to those of dyes, e.g., Pigment Red 151 (C.I. 15892) and Pigment Yellow 168 (C.I. 13960), which suggests the possibility of combining inorganic salts and dyes. Calcium carbonate is commonly used as a filler in the plastic industry because of its wide availability and low cost, as well as its special particle shapes [11]. Barium sulfate is resistant to acid and alkali, easy to disperse, and often used as a white pigment for paint [12]. Calcium phosphate is used as a stabilizer in plastics [13]. They were used to hybridize Weak Acid Pink Red B (APRB, C.I. 18073), Mordant Blue 9 (MB, C.I.14855) and Acid Brilliant Blue 6B (ABB6B, C.I. 42660) (Figure 1). The hybrids formed were added to polypropylene (PP) as colorants and their performance: e.g., dispersion, coloring power, resistance to solvents, migration, thermal and UV irradiation were measured and evaluated. 

## 2. Results and Discussion

### 2.1. Removing Synthetic Dyes by *in situ* Hybridization with Inorganic Salts

**Figure 2:**
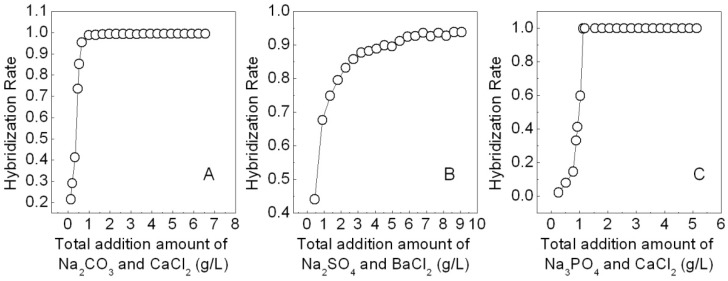
Effects of the addition amount of Na_2_CO_3_ and CaCl_2_ (molar ratio 1:2) on the hybridization of APRB (**A**), Na_2_SO_4_ and BaCl_2_ (molar ratio 1:1.5) on the hybridization of MB (**B**), and Na_3_PO_4_ and CaCl_2_ (the molar ratio 1:2) on the hybridization of ABB6B (**C**).

Three simulated dye effluents were treated with freshly prepared calcium carbonate, barium sulfate or calcium phosphate. The results indicated these salts all display high hybridization rates, *i.e.*, 99% for APRB, 94% for MB and 99.98% for ABB6B (Figure 2). It was found that the best molar addition ratio of APRB:Na_2_CO_3_:CaCl_2_ is 1:2:4 for which the hybridization rate of APRB reached 95%, suggesting that calcium carbonate has a great hybridization effect on APRB. The same phenomenon with a hybridization rate of 99.7% is observed for ABB6B when the molar addition ratio of ABB6B:Na_3_PO_4_:CaCl_2_ is 1:3:6. As for MB, the hybridization rate is 90% when the molar addition ratio of MB:Na_2_SO_4_:BaCl_2_ is 1:100:150. In order to improve the efficiency of hybridization of these dyes on a large scale and ensure full utilization of the dyes, the preparation of the hybrids used for the following tests and analyses adopted lower addition molar ratios of 1:10:20 for ABB6B: Na_3_PO_4_:CaCl_2_ and 1:160:320 for APRB:Na_2_CO_3_:CaCl_2_. To ensure the dye content in the BaSO_4_-MB hybrid, the molar ratio of MB:Na_2_SO_4_:BaCl_2_ was 1:35:52. After washing and drying, the dye contents in the CaCO_3_-APRB hybrid, the BaSO_4_-MB hybrid and the Ca_3_(PO_4_)_2_-ABB6B hybrid were determined by elemental analysis to be 4.8, 4.1 and 23.4%, respectively. In order to reuse these products after treating, characterization and application as colorant experiments were carried out.

### 2.2. Characterization of the Dye Hybrids

#### 2.2.1. CaCO_3_-APRB hybrid

**Figure 3:**
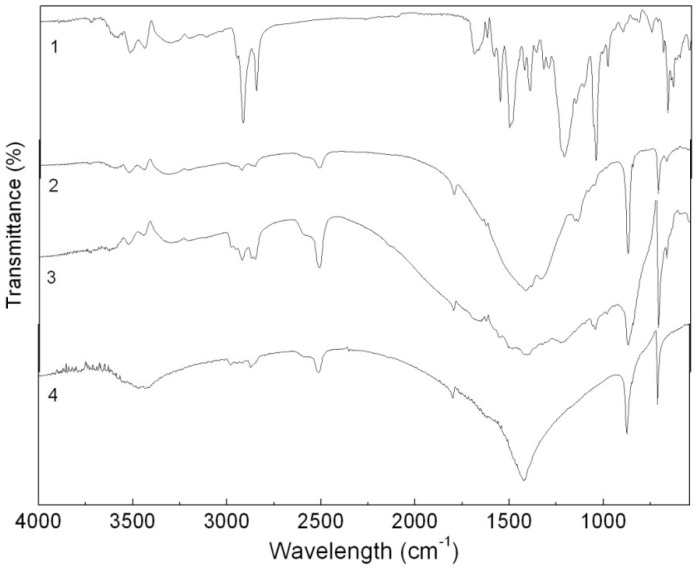
FTIR spectra of the APRB (**1**), the CaCO_3_-APRB hybrid (**2**), the CaCO_3_/APRB mixture (**3**) and CaCO_3_ (**4**).

In the IR spectrum of the CaCO_3_-APRB hybrid (Figure 3), a strong and wide peak at 1417 cm^−1^, a moderately strong peak at 873 cm^−1^ and a small peak at 712 cm^−1^ are assigned to the characteristic vibration bands of CaCO_3_, namely the CaCO_3_ ν(CO_3_^2−^) stretching vibration band, the CO_3_^2−^ bending and rocking vibration bands and the Ca-O stretching and bending vibrations [8]. In addition, the characteristic IR adsorption bands of APRB could also be observed, *i.e.*, the band from 3000 to 3600 cm^−1^ for N-H, C-H and O-H stretching vibrations, and 2926 and 2856 cm^−1^ for -CH_2_ asymmetric stretching vibration and symmetric vibration. However, the sulfonic group IR adsorption peak at around 1216 cm^−1^ isn’t detected, as it is overlapped by a broad CO_3_^2−^ vibration band around 1400 cm^−1^. It is reported that noncovalent interactions fix APRB and Ca^2+^ between the temporary electric double layer of CaCO_3_ forming an onion-like structure hybrid [8]. Unlike the hybrid, the IR spectrum of the CaCO_3_/APRB mixture showed all the characteristic vibrations of the two compounds in the same positions, so we can conclude that no reaction occurred between CaCO_3_ and APRB in the mixture form.

**Figure 4:**
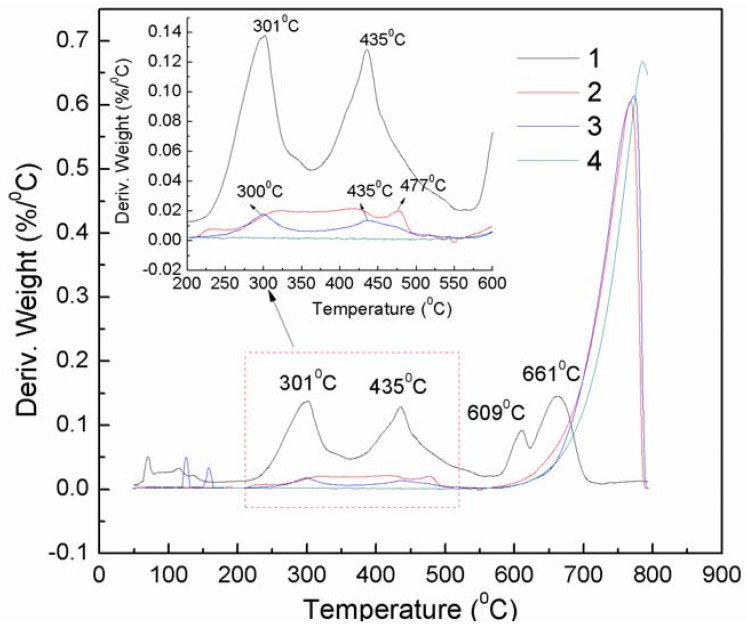
DTG curves of powders APRB (**1**), the CaCO_3_-APRB hybrid (**2**), the CaCO_3_/APRB mixture (**3**) and CaCO_3_ (**4**).

The DTG analysis also verifies the above results. APRB shows three major weight losses at 301, 435 and 661 °C (Figure 4). 

The first and second weight losses are attributed to the decomposition of the −C_12_H_25_ alkyl chain and the azo naphthol amide. The third weight loss is caused by two sulfonic groups that could be transformed into sodium sulfate [8]. The CaCO_3_/APRB mixture presents the same weight loss peaks at 301 and 435 °C as APRB, suggesting that the thermal stability of APRB doesn’t change below 550 °C, but the CaCO_3_-APRB hybrid has a broad weight loss peak between 275 and 500 °C, which has the same start and end positions as APRB. Thus, there is an intermolecular interaction between APRB and CaCO_3_. In this way, the combination of APRB and CaCO_3_ in the hybrid is stronger and more homogeneous than in the mixture, which results in their distinct performance as colorant filler in PP.

#### 2.2.2. The BaSO_4_-MB hybrid

**Figure 5:**
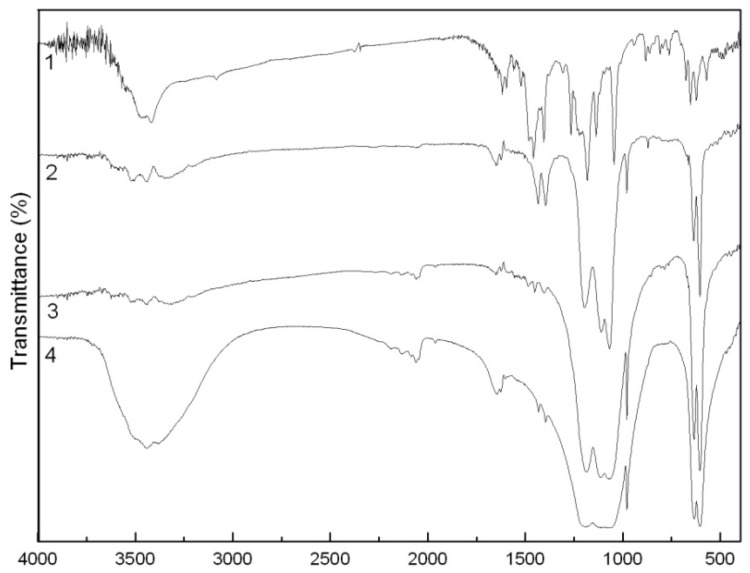
FTIR spectra of the MB (**1**), the BaSO_4_-MB hybrid (**2**), the BaSO_4_/MB mixture (**3**) and BaSO_4_ (**4**).

In the FTIR spectrum of the BaSO_4_-MB hybrid (Figure 5), strong vibration bands at 1200 and 1074 cm^−1^, similar with those of BaSO_4_, are ascribed to the characteristic IR absorption peaks of SO_4_^2−^ [14]. The characteristic IR absorption bands of MB are also observed in the spectrum of the hybrid. For example, the MB bands from 3000 to 3600 cm^−1^ for O-H and C-H stretching vibrations at 3527, 3448 and 3317 cm^−1^ correspond with the bands in the same positions of the BaSO_4_-MB hybrid. The broad band from 1000 to 1350 cm^−1^ in the hybrid has three sharp peaks, which correspond to characteristic SO_4_^2−^ peaks of, being different from the broad peaks in the BaSO_4_/MB mixture or in BaSO_4_. The O-H peaks at 1438 and 1400 cm^−1^ in the hybrid are isolated from the broad band of SO_4_^2−^ or –SO_3_^−^ from 1000 to 1350 cm^−1^, which is close to MB. In this way, we can conclude that the combination of BaSO_4_ and MB in the hybrid is not a physical mixture. It is reported that BaSO_4_ and MB presents electrostatic interactions in the formation of the hybrid [15]. As for the BaSO_4_/MB mixture, nearly all the characteristic IR adsorption peaks of MB and BaSO_4_ appear the same positions and shapes as their single materials, suggesting that no interaction is existed. 

**Figure 6:**
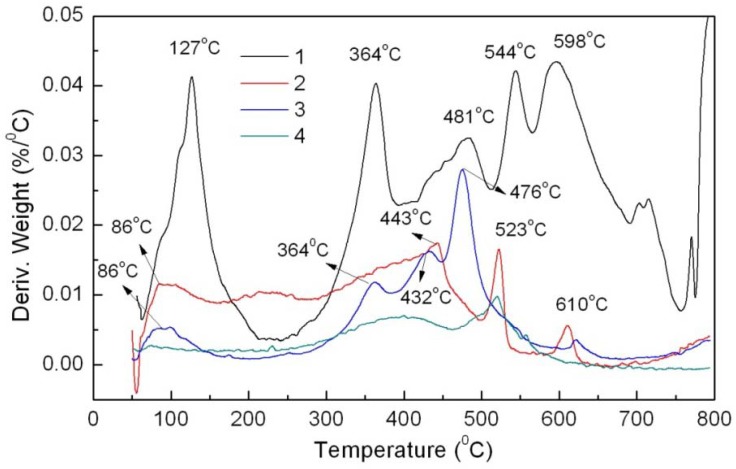
DTG curves of powder MB (**1**), the BaSO_4_-MB hybrid (**2**), the BaSO_4_/MB mixture (**3**), and BaSO_4_ (**4**).

DTG of the four materials were analyzed. The weight loss of the BaSO_4_-MB hybrid (2.47%) is more than that of the BaSO_4_-MB mixture (0.85%) under 200 °C (Figure 6). This might imply poor thermal stability when used as plastic additive. The BaSO_4_/MB mixture has three weight loss peaks at 364, 432 and 476 °C, being similar to MB between 300 °C and 500 °C. However, the BaSO_4_-MB hybrid has two weight loss peaks at 523 and 610 °C, and a broad weight loss peak appeared from 100 to 450 °C, being different from MB. This indicates an interaction between MB and BaSO_4_, which is similar to the DTG curve of the CaCO_3_-APRB hybrid, showing a same result as the FT-IR analysis. The hybrid has the same content of MB and BaSO_4_ as the BaSO_4_/MB mixture, but its DTG curve is above that of the mixture in the temperature region from 70 to 500 °C. That means the BaSO_4_-MB hybrid has a greater weight loss than the BaSO_4_/MB mixture, and it is more sensitive to temperature than the BaSO_4_/MB mixture.

#### 2.2.3. The Ca_3_(PO_4_)_2_-ABB6B hybrid

**Figure 7:**
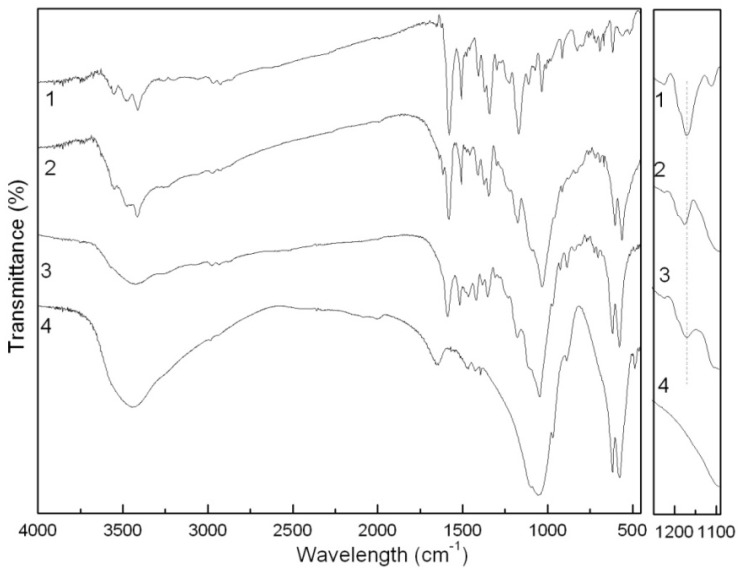
FTIR spectra of the ABB6B (**1**), the Ca_3_(PO_4_)_2_-ABB6B hybrid (**2**), the Ca_3_(PO_4_)_2_/ABB6B mixture (**3**) and Ca_3_(PO_4_)_2_ (**4**).

In the FTIR spectrum of the Ca_3_(PO_4_)_2_-ABB6B hybrid (Figure 6), characteristic ABB6B and Ca_3_(PO_4_)_2_ adsorption peaks can be found, e.g., the -CH_2_ vibration peaks of ABB6B at 2975 and 2926 cm^−1^, benzene ring ones at 1581 and 1508 cm^−1^, –CH_3_ at 1374 cm^−1^, C-N at 1346 cm^−1^, and -SO_3_^2^ at 1176 cm^−1^, and the PO_4_^3−^ vibration peaks of Ca_3_(PO_4_)_2_ at 1032, 603 and 562 cm^−1^ [16]. These characteristic adsorption peaks of ABB6B and Ca_3_(PO_4_)_2_ can also be observed in the Ca_3_(PO_4_)_2_/ABB6B mixture. However, differences exist between the hybrid and the mixture. As seen in Figure 7, the adsorption peak of –SO_3_^−^ in ABB6B at 1170 cm^−1^ is shifted to a higher frequency of 1176 cm^−1^ in the hybrid, which might be attributed to some electrostatic interaction between –SO_3_^−^ and Ca^2+^. The phenomenon isn’t seen in the mixture, so we conclude that the hybrid’s structure is different from that of the mixture. 

**Figure 8:**
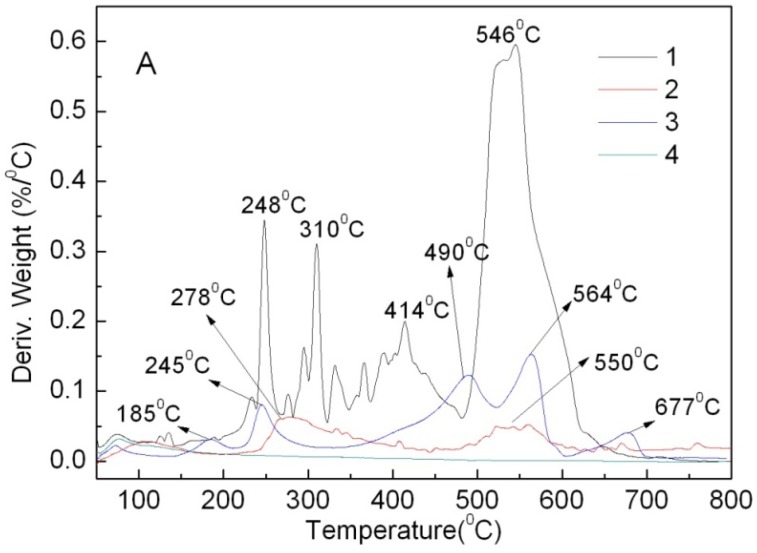
DTG curves of ABB6B (**1**), the Ca_3_(PO_4_)_2_-ABB6B hybrid (**2**), the Ca_3_(PO_4_)_2_/ABB6B mixture (**3**) and Ca_3_(PO_4_)_2_ (**4**).

From The DTG curves in Figure 8, ABB6B has four major weight loss peaks at 248, 310, 414 and 546 °C and the Ca_3_(PO_4_)_2_-ABB6B hybrid two peaks at 278 and 550 °C. 

The two weight loss peaks at 310 and 414 °C are not found in curve 2, and the first peak at 248 °C for ABB6B shifts to 278 °C for the hybrid. Thus, the combination of ABB6B with Ca_3_(PO_4_)_2_ improves the thermal stability of ABB6B. Curve 3 for the Ca_3_(PO_4_)_2_/ABB6B mixture exhibits five major weight losses at 185, 245, 490, 564 and 677 °C. Two peaks have the similar positions to those of ABB6B at 248 and 546 °C. Curve 3 from 260 to 500 °C is different from that of ABB6B and it shows less weight loss peaks than ABB6B. The phenomenon is different from that of the CaCO_3_/APRB and BaSO_4_/MB mixtures. These phenomena of less weight loss peaks and delayed weight loss peaks are due to the special properties of Ca_3_(PO_4_)_2_. From IR curve 2 in Figure 8, an OH absorption is found in the Ca_3_(PO_4_)_2_-ABB6B hybrid. Thus, the thermal decomposition of the hybrid might result in the formation of phosphoric acid. The formations of carbonized residues with phosphate insulate the ABB6B underneath from heat [17]. Moreover, the weight loss (only 20.57%) of the Ca_3_(PO_4_)_2_-ABB6B hybrid is less than that of the Ca_3_(PO_4_)_2_/ABB6B mixture (27.79%) before 800 °C. Therefore, the Ca_3_(PO_4_)_2_-ABB6B hybrid exhibits a thermal stability which shows better performance than the Ca_3_(PO_4_)_2_/ABB6B mixture.

### 2.3. Application of the Hybrids as Colorants

#### 2.3.1. Resistance to solvents

**Figure 9:**
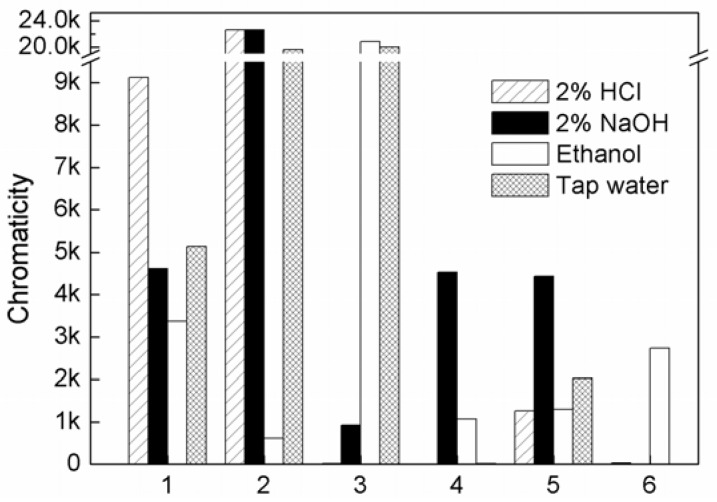
Color release of APRB (**1**), MB (**2**), ABB6B (**3**), the CaCO_3_-APRB (**4**), BaSO_4_-MB (**5**) and Ca_3_(PO_4_)_2_-ABB6B (**6**) hybrids dissolved in 2% HCl, 2% NaOH, ethanol and tap water.

If a dye is applied as a plastic colorant, color migration will be not allowed. These hybrids with dyes are insoluble, which may improve the dye resistance to solvent. Four kinds of media e.g., neutral tap water, acidic solution (2% HCl), alkaline solution (2% NaOH) and ethanol were used to examine the color release of the hybrids (Figure 9). 

The colorations of the supernatant of the CaCO_3_-APRB hybrid liquid in 2% NaOH, ethanol and tap water are much less than that of APRB-only (Figure 9, 1/4). The release of MB from the BaSO_4_-MB hybrid in 2% HCl, 2% NaOH and tap water is much less than that of MB-only (Figure 9, 2/5). ABB6B is soluble in ethanol and tap water, hardly soluble in 2% HCl and 2% NaOH (Figure 9, 3). However, the Ca_3_(PO_4_)_2_-ABB6B hybrid is insoluble in 2% HCl, 2% NaOH and tap water (Figure 9, 6). Thus, these hybrids obviously improve the dyes’ resistance to various solvents, particularly in the case of the Ca_3_(PO_4_)_2_-ABB6B hybrid.

#### 2.3.2. Dispersion of colorants added into PP samples

**Figure 10:**
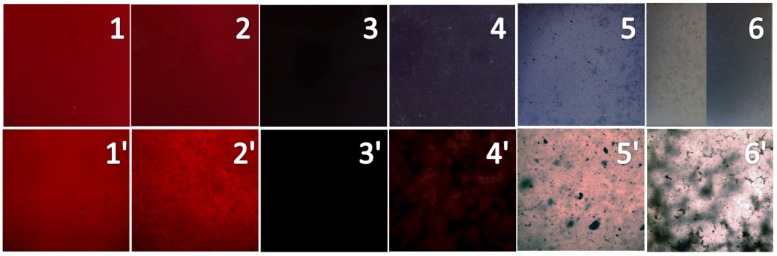
Photographs of PP samples colored with the CaCO_3_-APRB hybrid (**1**), the CaCO_3_/APRB mixture (**2**), the BaSO_4_-MB hybrid (**3**), the BaSO_4_/MB mixture (**4**), the Ca_3_(PO_4_)_2_-ABB6B hybrid (**5**), and the Ca_3_(PO_4_)_2_/ABB6B mixture (**6**). 1-6 are pictures of PP samples, 1′-6′ are images (×100) of the PP samples under the microscope.

The CaCO_3_-APRB hybrid, CaCO_3_/APRB mixture, BaSO_4_-MB hybrid and BaSO_4_/MB mixture as colorants added into PP bring the homogeneous dispersion and good coloration after kneading and molding at 200 °C (Figure 10, 1-4). The Ca_3_(PO_4_)_2_-ABB6B hybrid and the Ca_3_(PO_4_)_2_/ABB6B mixture show poorer dispersion but the former exhibits a better coloration than the latter (Figure 9, 5, 6). A majority of the colorants keep their colors unchanged during the processing. As an exception, the addition of the purple BaSO_4_-MB hybrid and the blue Ca_3_(PO_4_)_2_/ABB6B mixture make the PP samples dark reddish-brown and light blue. Moreover, the Ca_3_(PO_4_)_2_/ABB6B mixture forms a seriously inhomogeneous colour (Figure 9, 6). This is ascribed to the poorer thermal stability of the Ca_3_(PO_4_)_2_/ABB6B mixture than the Ca_3_(PO_4_)_2_-ABB6B hybrid. In addition, the CaCO_3_-APRB and Ca_3_(PO_4_)_2_-ABB6B hybrids present more brilliant colors than the CaCO_3_/APRB and Ca_3_(PO_4_)_2_/ABB6B mixtures.

In order to further investigate the dispersion of the colorants, the colored PP samples were observed with microscope (Figure 10, images 1′ to 6′). Little speckles appear in the PP samples colored with the CaCO_3_/APRB mixture, and flocci in PP with the BaSO_4_/MB mixture. Both the CaCO_3_-APRB and BaSO_4_-MB hybrids exhibit homogeneous dispersions. Thus, the hybridization of dye into CaCO_3_ or BaSO_4_ could give a better dispersion than their mixtures. The BaSO_4_-MB hybrid presents a much deeper color than the BaSO_4_/MB mixture and other colorants. Thus, the BaSO_4_-MB hybrid exhibits a strong covering power. Many little speckles were distributed on the PP samples colored with the Ca_3_(PO_4_)_2_-ABB6B hybrid (Figure 10, 5′). However, the Ca_3_(PO_4_)_2_/ABB6B mixture causes flocci and speckles (Figure 10, 6′). The hybridization of ABB6B into Ca_3_(PO_4_)_2_ can improve the dispersion of ABB6B. All of these hybrids show better dispersions than their mixtures, which matches the previously reported results [5,7].

#### 2.3.3. Migration of colorants from PP

**Figure 11:**
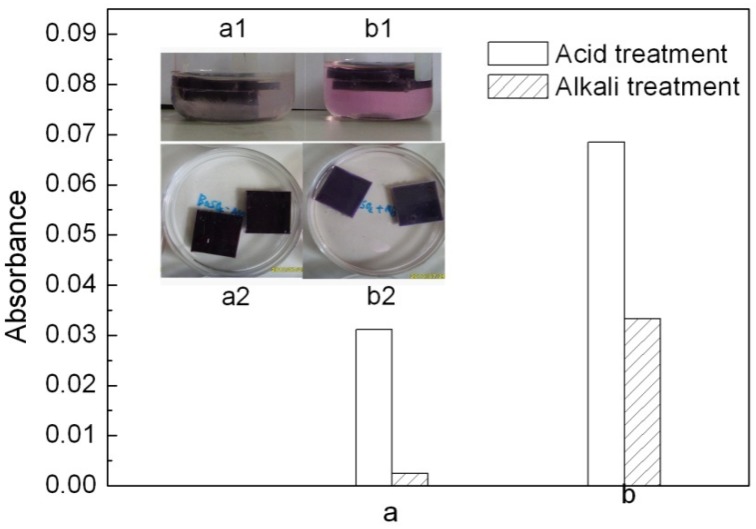
Change of the PP samples colored with the BaSO_4_-MB hybrid **(a)** and the BaSO_4_/MB mixture **(b)** when immersed in 2% NaOH (**a1, b1**) and 2% HCl (**a2, b2**). a and b: change of absorbance of the solvents determined at 523 nm for MB by spectrophotometry.

Migration of a colorant toward adjacent plastic or solvent is an important factor to evaluate a colorant’s application [2]. The colored PP samples were immersed in acid (2% HCl) and alkaline (2% NaOH) solutions. The CaCO_3_-APRB hybrid, CaCO_3_/APRB mixture, Ca_3_(PO_4_)_2_-ABB6B hybrid and Ca_3_(PO_4_)_2_/ABB6B mixture weren’t extracted from their PP samples. However, the migration of MB occurred from the PP samples colored with the BaSO_4_-MB hybrid and BaSO_4_/MB mixture (Figure 11 a1, b1). From the absorbance change of the media (Figure 11 a,b), the BaSO_4_-MB hybrid exhibits a better resistant to migration than the BaSO_4_/MB mixture. The MB migration from the BaSO_4_/MB mixture was 2 times higher than that of the BaSO_4_-MB hybrid in alkaline solution, and 12 times as high as the hybrid in acid solution. Thus, the BaSO_4_-MB hybrid shows a better migration resistance than their mixture.

#### 2.3.4. Thermal stability of colorants added into PP

**Figure 12:**
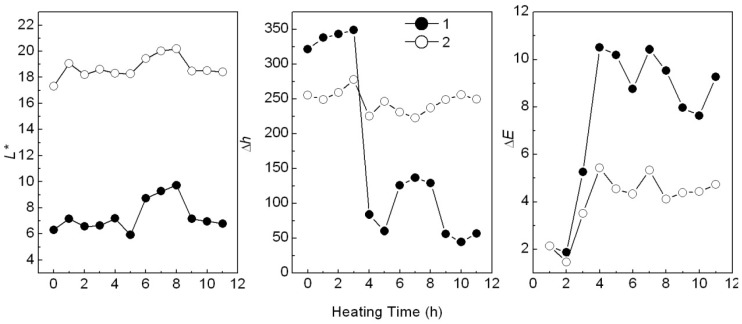
Effect of heating time on L*, h and ΔE of the PP samples colored with the BaSO_4_-MB hybrid (**1**) and the BaSO_4_/MB mixture (**2**) at 100 °C.

When added in plastic, a colorant may change color after heating. For example, the Ca_3_(PO_4_)_2_/ABB6B mixture changed from blue to light blue or light yellowish-brown during plastic processing. The PP sample colored with the CaCO_3_-APRB hybrid was hardly affected by heating. The color change (Δ*E*) of the CaCO_3_-APRB hybrid was less than 3 after heating for 11 h at 100 °C, being similar to that of the CaCO_3_/APRB mixture (Figure S2). As heating time was increased, the BaSO_4_-MB hybrid exhibited an obvious color change, *i.e.*, 10.5 of Δ*E* after heating for 3 h, resulting from the color tone Δ*h* > 100° and ΔL* < 4 (Figure 12). The change of hue was much greater than that of L*, so we can conclude that the color difference mainly comes from hue. This change due to hue rather than fading (caused by L*) would suggest that BaSO_4_-MB hybrid might have potential use as a heat-variable indicator. This performance is attributed to its thermal instability. The hybrid has a broad weight loss peak from 86 to 450 °C (Figure 6). The temperature in the heating test is 100 °C which resulted in color change of the hybrid. However, the BaSO_4_/MB mixture exhibited a less change of color tone (Δ*h* < 30°) and the color difference (Δ*E* < 6).

**Figure 13:**
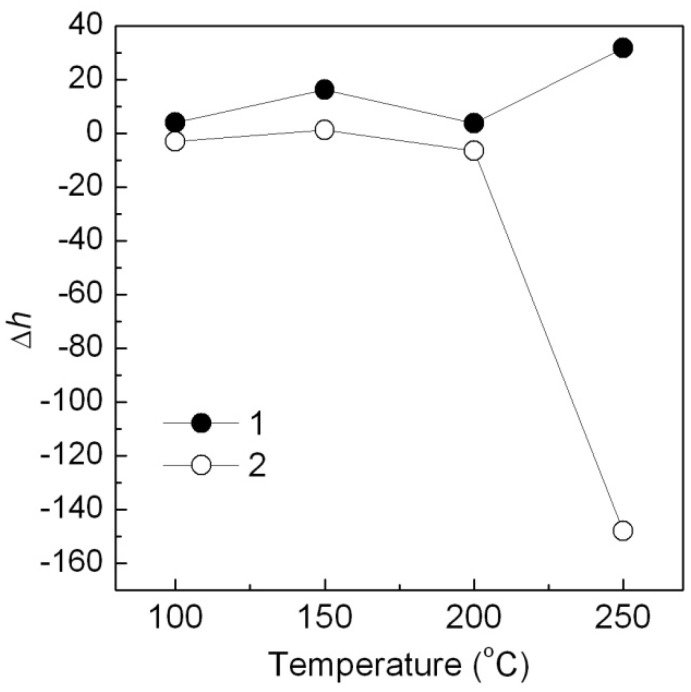
Effect of different temperatures on Δh of the Ca_3_(PO_4_)_2_/ABB6B hybrid (**1**), the Ca_3_(PO_4_)_2_-ABB6B mixture (**2**) and in PP for 1h of heating.

With the same method, the Ca_3_(PO_4_)_2_-ABB6B hybrid shows a better thermal stability than the Ca_3_(PO_4_)_2_/ABB6B mixture when added into PP. It may be ascribed to the heat absorption of Ca_3_(PO_4_)_2_ [18]. From effect of temperature (Figure 13), *h* values of the PP samples colored with the Ca_3_(PO_4_)_2_-ABB6B hybrid and the Ca_3_(PO_4_)_2_/ABB6B mixture hardly changed below 200 °C. 

However, Δ*h* of the mixture at 250 °C was much more than that of the hybrid, where the color of the Ca_3_(PO_4_)_2_/ABB6B mixture turned from blue to white. The Ca_3_(PO_4_)_2_-ABB6B hybrid exhibits a much better thermal stability than their mixture. That is consistent with the DTG analysis results.

#### 2.3.5. Photostability of the colorants added into PP

**Figure 14:**
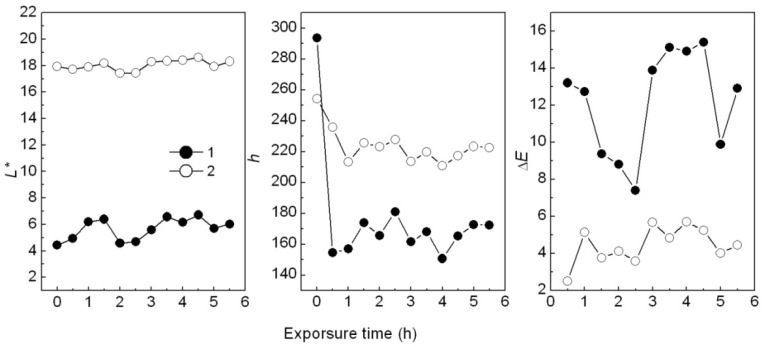
Effect of UV irradiation time on L* (**A**), h (**B**) and *ΔE* (**C**) of PP samples colored with the BaSO_4_-MB hybrid (**1**) and the BaSO_4_/MB mixture (**2**) at room temperature.

The photostability of hybrids was tested by UV irradiation when they were added into the PP samples. Results showed that the CaCO_3_-APRB hybrid colored in PP sample was hardly influenced, being similar to the CaCO_3_/APRB mixture (Figure S3). After UV irradiation for 0.5 h, the BaSO_4_-MB hybrid showed a serious color change, Δ*E* of 13.8 (Figure 14A). The color change mainly comes from hue change (Δ*h* = 139°), not fading (ΔL* = 0.5). With increased UV irradiation up to 1 h, the hue and brightness (L*) remains almost constant. From Figure 14 C, the Δ*E* of BaSO_4_/MB mixture had only a small change. Therefore, the hybrid could be recycled as a sensitive UV irradiation indicator, *i.e.*, reminding people about the solar radiation. 

**Figure 15:**
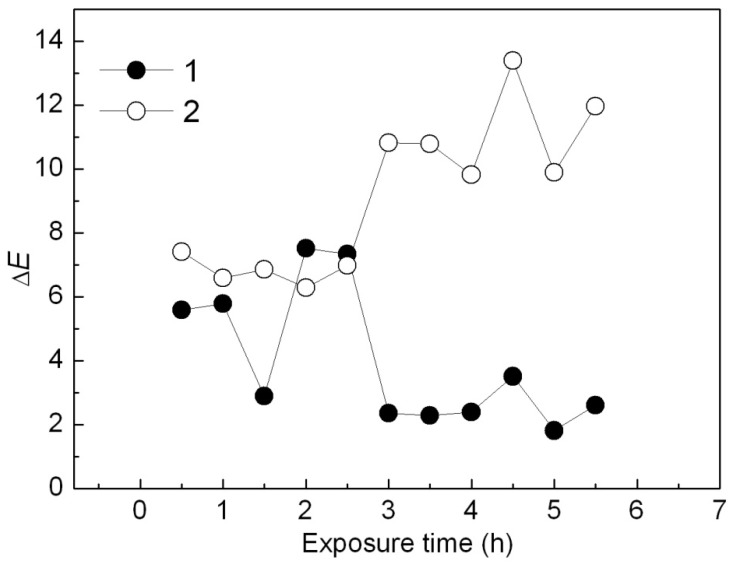
Effect of UV irradiation time on *ΔE* of PP samples colored with the Ca_3_(PO_4_)_2_-ABB6B hybrid (**1**) and the Ca_3_(PO_4_)_2_/ABB6B mixture (**2**).

From curve a in Figure 15, Δ*E* value of the Ca_3_(PO_4_)_2_-ABB6B hybrid decreased from 5.6 to 2 in the first 3 h and remained at 2 with increase of the UV exposure time. However, Δ*E* of the Ca_3_(PO_4_)_2_/ABB6B mixture increased obviously from 7 to 12 after 3 h from curve 2 in Figure 14. Thus, the Ca_3_(PO_4_)_2_-ABB6B hybrid caused little color difference when exposed to UV. The PP sample colored with the Ca_3_(PO_4_)_2_-ABB6B hybrid shows a better UV light stability. 

#### 2.3.6. Crystallization of the PP composites

The incorporation of inorganic fillers in PP can change the crystallization process and these changes can influence mechanical properties of PP [19]. Thus, the crystallization of the PP composites was investigated by FTIR and DSC. The level of crystallinity of PP is enhanced with the incorporation of these hybrids (Table 1). 

**Figure 16:**
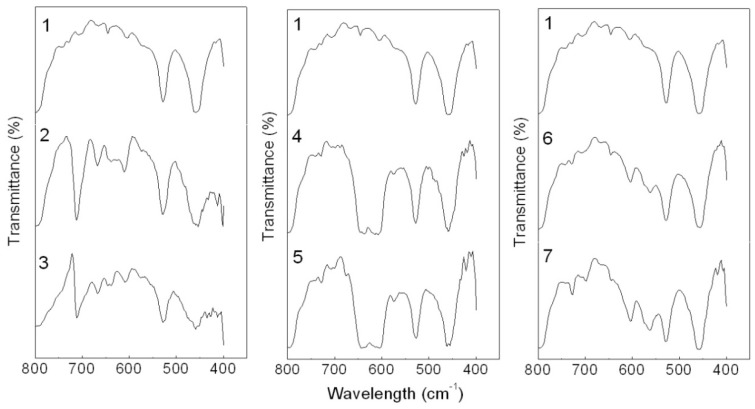
FTIR spectra of PP composites containing neat PP (1), the CaCO_3_-APRB hybrid (2), the CaCO_3_/APRB mixture (3), the BaSO_4_-MB hybrid (4), the BaSO_4_/MB mixture (5), the Ca_3_(PO_4_)_2_-ABB6B hybrid (6) and the Ca_3_(PO_4_)_2_/ABB6B mixture (7).

**Figure 17:**
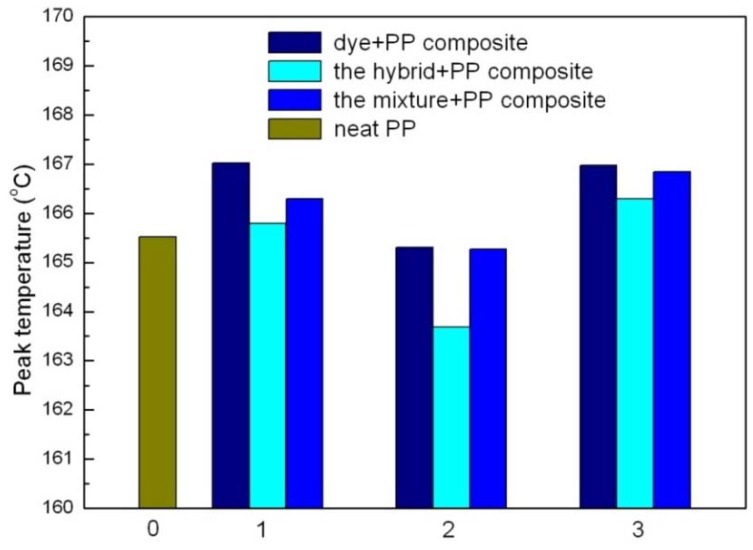
Temperature of different filler compositions in PP. 0 for neat PP, 1 for APRB related PP composites; 2 for MB related PP composites; 3 for ABB6B related PP composites.

Among them, the CaCO_3_-APRB and the Ca_3_(PO_4_)_2_-ABB6B hybrids have similar effects on the crystallinity of PP as nano- CaCO_3_ and nano- Ca_3_(PO_4_)_2_. As for the BaSO_4_-MB hybrid, it shows a greater effect on the crystallinity of PP than reported for BaSO_4_. This indicates that a nucleation effect of the hybrids as fillers might exist in the matrix crystallization process. It can also be observed in the FTIR curves (Figure 16). New peaks are recorded at 699 and 688 cm^−1^ for the BaSO_4_-MB composite, 699 and 563 cm^−1^ for the Ca_3_(PO_4_)_2_-ABB6B composite. In the case of CaCO_3_-APRB composite, a broad peak appears from 593 to 652 cm^−1^ which is different from the PP composite with CaCO_3_/APRB mixture, suggesting that some interaction between the CaCO_3_-APRB hybrid and PP occurred during the process. The peak at 605 cm^−1^ for PP shifts to higher frequency of 611 cm^−1^ for CaCO_3_-APRB composite. All these results show that the hybrids affect the crystallization process of PP. In addition, all the hybrids present lower melting temperature than their mixtures and related dyes, indicating that the hybrids change the effects of their mixtures and these dyes on the crystallization of PP (Figure 17). Among of them, the BaSO_4_-MB composite exhibits lower melting temperature than neat PP, but higher crystallinity than neat PP. It demonstrates that the BaSO_4_-MB hybrid is benefit to decrease processing temperature while obtain high mechanical properties of PP composite, which might be very useful to application of PP.

## 3. Experimental

### 3.1. Preparation of Dye-Inorganic Salt Material

An APRB (1.02 g) solution was mixed thoroughly with Na_2_CO_3_ (10.60 g) solution, and then CaCl_2_⋅2H_2_O (29.41 g) solution was added into the mixture slowly. After stirring for 30 min, the suspending substance was precipitated, washed, dried at 105 °C for 3 h and milled into powder (identified as the CaCO_3_-APRB hybrid). By the same method, the BaSO_4_-MB and the Ca_3_(PO_4_)_2_-ABB6B hybrids were synthesized, where MB (0.73 g), Na_2_SO_4_ (14.20 g) and BaCl_2_⋅2H_2_O (17.50 g), or ABB6B (6.78 g), Na_3_PO_4_⋅12H_2_O (38.01 g) and CaCl_2_⋅2H_2_O (29.41 g) replaced APRB, Na_2_CO_3_ and CaCl_2_⋅2H_2_O, respectively. The dye contents in the hybrids were determined by elemental analysis (Vario EL III, German). The thermal gravimetric analysis (TGA) and Fourier Transform Infrared analysis (FTIR) of the powder were carried using a thermogravimetric system (Model TAQ 600, USA) and an infrared spectrometer system (Model Equinoxss/hyperion 2000, Germany), respectively.

### 3.2. Anti-Solvency of Dye Hybrids as Colorants

The CaCO_3_-APRB hybrid powder was dispersed in 2% HCl, 2% NaOH, ethanol and tap water and mixed thoroughly for 30 min. The liquid was centrifuged and the coloration of the supernatant determined with a spectrometer (Model S4100, Scinco, Korea) with the Labpro Plus software (Firmware Version 060105). According to the same method, the anti-solvency experiments of the BaSO_4_-MB and the Ca_3_(PO_4_)_2_-ABB6B hybrids were carried out.

### 3.3. Preparation of PP Samples with Characterization

**Figure S1:**
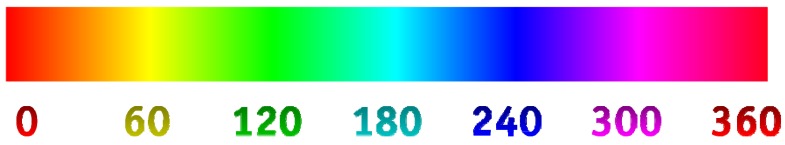
Hue in the HSB/HSL encodings of RGB.

**Figure S2:**
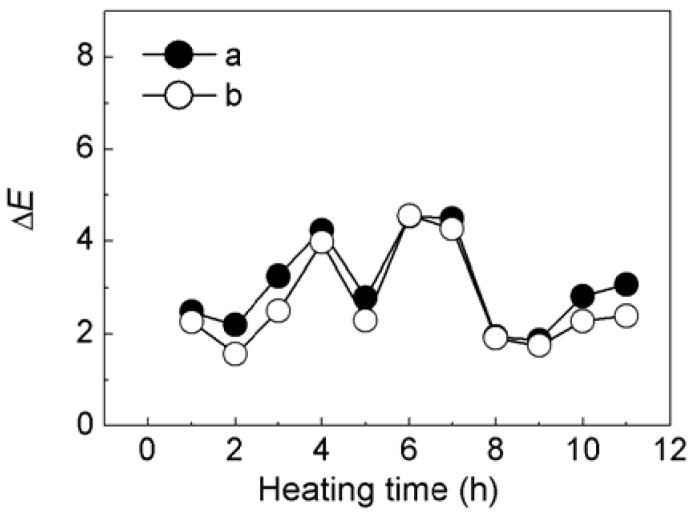
Effect on heating time on *ΔE* of colorants of CaCO_3_-APRB hybrid (a) and CaCO_3_-APRB mixture (b) in PP at 100 °C.

**Figure S3:**
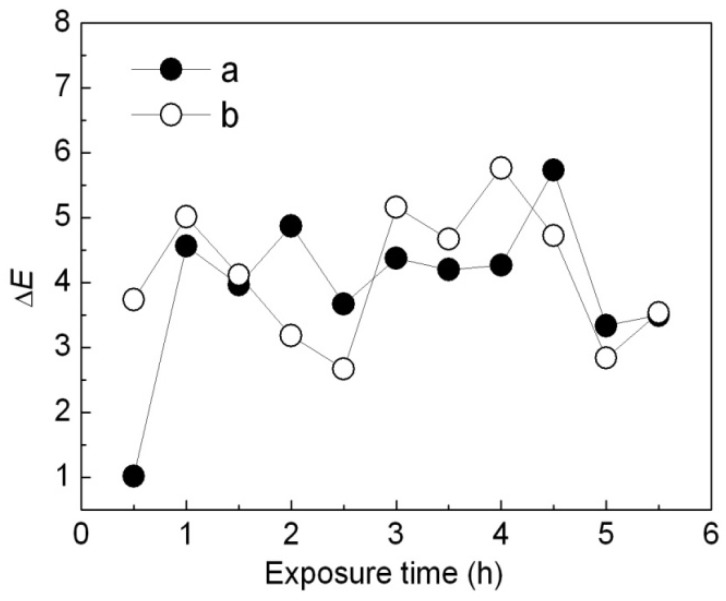
Effect of UV irradiation time on ΔE of colorants of CaCO_3_-APRB hybrid (a) and CaCO_3_-APRB mixture (b) in PP.

The hybrid was used as colorant to mix with 200 g of PP. The resulting mixture was put in a kneader at various temperatures which included a mixing tank of 1,500 cm^3^ and counter rotating roller blades. The colored PP samples were molded in 30 × 30 × 2 mm at around 200 °C using a Model QL20 molding machine (Hangzhou, China). The molded PP samples were measured with a Model WSC –Y automatic colorimeter (Beijing, China) and the dispersion of dye observed with a microscope. The color difference meter was used to determine L* (lightness-darkness), a* (redness-greenness) and b* (yellowness-blueness) (CIELAB) of the colored PP samples. Changes of the color difference (Δ*E*), hue angle (*h*) and chroma (C*_ab_) were calculated by the following relationships [21]:

(1) $$\Delta E=\sqrt{{\left(L_{1}^{*}-L_{0}^{*}\right)}^{2}+{\left(a_{1}^{*}-a_{0}^{*}\right)}^{2}+{\left(b_{1}^{*}-b_{0}^{*}\right)}^{2}}$$

(2) $$h=\tan^{-1}(b^{*}/a^{*})$$

(3) $$\Delta h=h_{1}-h_{0}$$

(4) $$C_{\mathrm{ab}}^{*}=\sqrt{{\left(a^{*}\right)}^{2}+{\left(b^{*}\right)}^{2}}$$

where 1 and 0 refer to the reference and the test specimen. Δ*E* can be correlated with the colour differences in their visually perceived colour. However, it doesn’t indicate direction of hue, chroma, and lightness differences. Both *h* and C*_ab_ are used to judge the direction of the color difference. The *h* value changes from 0 to 360°, as seen in Figure S1 [22]. In the CIELAB color system, the absolute magnitude of color change between two conditions is given by Δ*E*. An Δ*E* value of one unit is approximately equivalent to a color difference that is just visually perceptible to 50% of observers under controllable conditions. Value of Δ*E* from 2 to 3 represents the color difference that are slightly perceptible, and more than 3.3 is visually perceptible to 50% of observers [23]. Δ*E* more than 7 shows marked color difference [24]. 

The crystallinity of virgin PP and its composites was determined with the following relationship:

(5) $$\mathrm{Crystallinity}\left(\%\right)=\frac{\Delta H_{f}}{\Delta H_{s}\times X_{\mathrm{PP}}}$$

Where ∆*H_f_* is the heat of fusion of the PP polymer and ∆*H_s_* is the heat of fusion of PP under standard conditions (*i.e.*, 208 J/g [19]), *X_pp_* is the weight percentage content of PP in the PP composites.

### 3.4. Migration, Thermal Stability and Photostability of Colorants

For the migration test, the colored PP samples were soaked in 2% HCl and 2% NaOH for 18 h. The absorbances of the solvents were measured by spectrophotometry. For thermal stability tests, the colored PP samples were put into an oven for 1 h at 100, 150, 200 and 250 °C, respectively, or put into an oven at 100 °C for 1 h × 11 times. The color parameters of the samples after testing were determined with the automatic colorimeter mentioned above. For photostability tests, the colored PP samples were irradiated 11 times with a UV lamp (300 w) in an enclosed wooden box (30 min). The color parameters of the samples were then determined. 

## 4. Conclusions

The acidic dyes APRB, MB and ABB6B were hybridized with three insoluble inorganic compounds, calcium carbonate, barium sulfate and calcium phosphate. As colorants, the formed hybrids exhibit good anti-solvency effects, good dispersion, brilliant and uniform color, and high resistance to migration when added to PP. Besides, the BaSO_4_-MB hybrid causes an obvious color change under thermal and UV irradiation treatment and it may be a potential heat/UV indicator. The PP sample colored with the Ca_3_(PO_4_)_2_-ABB6B hybrid displays a controllable change over long periods under UV light exposure, and might be used as a photostabilizer. The hybridization of Ca_3_(PO_4_)_2_ with dye would improve the dye’s thermal stability. These hybrids show the same effects on crystallinity of PP as related nano-inorganic materials. Especially the BaSO_4_-MB hybrid could decrease the processing temperature while maintain high crystallinity in comparison with neat PP. 

The hybridization of dyes with inorganic salts by the presented *in situ* synthetic method may improve the performance of dyes as plastic colorants. In this way, the hybrids can even replace heavy metal colorants in some fields and reduce pollution risks. It is reported that sludge obtained from dye wastewater by hybridization shows better performance as colorant than the sludge without hybridization [25]. In this way, if a dye wastewater replacing dye product is hybridized with these inorganic salts and then the hybridized sludge can be reused as colorant, both wastewater treatment and colorant production will be performed simultaneously.

## Figures and Tables

**Table 1:** Crystallinity for different compositions in PP.

Composition	Addition amount	Crystallinity	Relative neat PP crystallinity	Source
	wt %	%	%	
CaCO_3_-APRB hybrid	2.1	34.26	32.32	Self prepared
BaSO_4_-MB hybrid	2.4	38.52	32.32	Self prepared
Ca_3_(PO_4_)_2_-ABB6B hybrid	0.4	33.93	32.32	Self prepared
nano CaCO_3_	3	40	38	[19]
nano Ca_3_(PO_4_)_2_	0.5	29.02	25.88	[18]
BaSO_4_	8	46.8	46.2	[20]
